# Validity of Three Commercial Devices for Recording Movement Velocity in the Bulgarian Split Squat

**DOI:** 10.5114/jhk/189365

**Published:** 2024-12-06

**Authors:** Zhili Chen, Kaifang Liao, Chris Bishop, Chao Bian, Yongming Li

**Affiliations:** 1 School of Athletic Performance, Shanghai University of Sport, Shanghai, China.; 2 School of Sports Health, Guangdong Vocational Institute of Sport, Guangzhou, China.; 3 Faculty of Science and Technology, London Sport Institute, Middlesex University, London, United Kingdom.; 4 China Institute of Sport Science, Beijing, China.

**Keywords:** unilateral movement, 3D motion capture system, VBT, training monitoring

## Abstract

This study aimed to assess the validity of three commercial devices in recording mean velocity (MV) and peak velocity (PV) during a unilateral resistance exercise. Eighteen strength-trained and healthy males performed repetitions of Bulgarian split squats at loads ranging from 40% to 90% of their one-repetition maximum. MV and PV were simultaneously recorded by GymAware, PUSH, My Lift and compared to Vicon for all repetitions. Concurrent validity was assessed through a linear mixed model, as well as mean difference (MD), mean absolute error (MAE) and Hedge’s g effect sizes. GymAware was found to be valid in MV (MD = −0.02 to −0.01 m/s, MAE = 0.02 to 0.03 m/s, g = −0.08 to −0.19) and PV (MD = 0.01 to 0.05 m/s, MAE = 0.05 to 0.07 m/s, g = −0.06 to −0.22) recordings. Significant differences were identified between GymAware, PUSH, My Lift and Vicon for both MV (p < 0.01) and PV (p < 0.01) assessments. Moreover, when comparing MV and PV recorded by PUSH and My Lift to Vicon, larger MD and MAE, and trivial to moderate effects were also evident. Therefore, our findings suggest that GymAware could be an alternative for recording MV and PV during unilateral resistance exercises.

## Introduction

In recent years, the concept of velocity-based training (VBT) has been given increased attention in both practice and research (González-Badillo et al., 2011; González-Badillo and Sánchez-Medina, 2010; Sánchez-Medina and González-Badillo, 2011). Compared with traditional resistance training approaches (i.e., prescribing loads from a percentage of a previously determined one-repetition maximum (Thompson et al., 2020)), the intensity of VBT can be controlled by monitoring the movement velocity (e.g., mean velocity (MV) and peak velocity (PV)) in real time (Cui et al., 2024), and adjusting training loads and volume according to target velocities (Randell et al., 2011; Rebelo et al., 2023; Weakley et al., 2019, 2020). Since minor differences (~0.07 m/s) in MV typically represent significant improvements (~5% 1RM) in main resistance exercises such as the bench press and the full back squat (Courel-Ibáñez et al., 2019), optimal implementation of VBT relies on the use of both reliable and valid devices in order to prevent the natural variation in performance from being masked by measurement error in the device itself.

Typically, 3D motion capture systems (e.g., Vicon, Qualysis), which use reflective markers to track the trajectory of a given movement, are regarded as the “gold standard” for recording movement velocity (Mitter et al., 2021; Randell et al., 2011; Weakley et al., 2021). However, the price and operational complexity hinder these devices from being used in the field. With the advancement of technology, more affordable devices have been developed and utilized in practice. For example, the GymAware PowerTool, utilizing an optical encoder and a retractable wire, effectively converts raw displacement data of the barbell into velocity, making it a viable replacement for a 3D motion capture system (Weakley et al., 2021), due to its strong reliability (Banyard et al., 2017; Thompson et al., 2020) and validity (Askow et al., 2018; Banyard et al., 2017; Thompson et al., 2020). Apart from this, portable and low-cost devices, such as wearable accelerometers and smartphone-based applications, are commonly preferred (Uysal et al., 2023). These devices have been instrumental in the promotion of VBT, making it available for every practitioner. However, they were shown to yield reduced reliability (Chéry and Ruf, 2019; Mcgrath et al., 2018) and validity (Banyard et al., 2017; Orange et al., 2019; Van den Tillaar and Ball, 2019) compared to linear position transducers (LPTs), such as the GymAware PowerTool.

Previous studies have explored the validity of commercial devices during bilateral exercises, such as the back squat (Pérez-Castilla et al., 2019; Weakley et al., 2021) and the bench press (Lake et al., 2019). However, unilateral exercises are also an important component of resistance exercise programs. Several studies have shown that unilateral exercises are comparable to bilateral exercises for improving performance during change of direction, jumping and sprinting tasks, while also being more effective for unilateral jump performance (Liao et al., 2022; Speirs et al., 2016; Szymczyk et al., 2022), and commonly being applied during resistance training programs (Bishop et al., 2022; Koyuncu et al., 2024; Liao et al., 2022). Nevertheless, considering the differences in movement patterns and force-generating muscle groups between unilateral and bilateral exercises, it is questionable whether the conclusions drawn from bilateral exercises can be extended to unilateral exercises. Hence, we recommend more information is needed related to commercial devices’ validity based on VBT, during unilateral exercises. Therefore, the aims of this study were to explore the validity of three commercial devices (e.g., GymAware, PUSH and My Lift) by concurrently comparing them to Vicon, during the Bulgarian split squat.

## Methods

### Participants

Eighteen strength-trained males who had been free from any lower extremity musculoskeletal injury within the last six months, volunteered to participate in this study. Participants were required to have at least two years of resistance training experience. Their strength levels were self-reported, and their 1RM values in the Bulgarian Split Squat are presented in Table 1. Prior to providing written informed consent, the aims and experimental procedures of the study, as well as the potential risks and benefits were explained to each participant. Ethical approval was granted by the Shanghai University of Sport (approval code: 102772021RT088; approval date: 21 March 2021).

**Table 1:** General information of study participants.

Age (yrs)	Body Height (cm)	Body Mass (kg)	1RM-BSS-L (kg)	1RM-BSS-R (kg)
21.1 ± 2.6	175.1 ± 6.0	70.4 ± 6.8	102.2 ± 12.4	104.2 ± 13.3

Notes: BBS = Bulgarian Split Squat

### Research Design

To assess the validity to devices, we recorded MV and PV using GymAware (GymAware Power Tool, Kinetic Performance Technologies, Canberra, Australia), PUSH Band 2.0 (PUSH Inc., Toronto, Canada), My Lift (Version 10.0.6 iOS) and a 3D motion capture system (Vicon motion systems, Oxford, UK) simultaneously, during the Bulgarian split squat on a Smith Machine (Lipper, Nantong, China). Participants were tested during three sessions. During the first session, participants were familiarized with the testing protocols and their range of motion (ROM) distances were recorded during the Bulgarian split squat exercise. After an interval of 24 h, all participants established their 1RM for the Bulgarian split squat during the second session. After a 48-h recovery period, a formal experiment was conducted, which included three repetitions at 40%, 50%, 60% and 70% of 1RM, and two repetitions at 80% and 90% 1RM. The left and right legs were tested in randomized order and sets were separated by a 5-min rest interval. MV and PV of each repetition were simultaneously recorded. All tests were performed under similar environmental conditions to ensure as much consistency as possible during testing.

### Procedures

#### 1RM Assessment

The Bulgarian split squat was performed following the standard technique as described in a previous study (Andersen et al., 2014). The test began with a standardized warm-up comprised of a 3-min jog and a series of dynamic stretches. After 5 min of rest, participants completed an incremental loading test to failure. The load started with 40 kg, and participants attempted to complete 3 to 5 repetitions. Then, 20 kg was added until the repetition MV < 0.7 m/s with three repetitions. Subsequently, 10 kg were added when MV ranged from 0.7 to 0.5 m/s, with two repetitions performed. Finally, when MV < 0.5 m/s was evident, increases of 1–5 kg were implemented until participants were able to achieve 1RM with a single repetition. If the attempt was successful, a 2- to 4-min rest interval was given, and more weight was added based on their own judgment until the participant could not complete one repetition. GymAware was adopted to collect MV for each repetition. For safety reasons, all test sessions and activities were performed on a Smith Machine (Lipper, Nantong, China). The Smith Machine consists of a rack that fully supports a regular Olympic barbell, to completely stabilize the barbell and allow a smooth, vertical movement of the barbell along a fixed path.

#### Bulgarian Split Squat

Participants started from an upright position and then separated their feet apart in a straight line from front to back, with the instep of the posterior foot placed on a special Bulgarian split squat stand set at a height of 40 cm (Helme et al., 2019). The heel of the anterior foot was required to stay in contact with the ground, and the distance between the anterior and posterior foot was the height of the tibial tuberosity in the upright position throughout the exercise. Meanwhile, participants held the barbell in a closed pronated grip and hands placed on the barbell slightly wider than shoulder width. The barbell was positioned on the upper trapezius muscle. Participants were instructed to control the descent (eccentric phase), lasting ~1–2 s until the top of the thigh of the front leg was parallel to the floor (forward knee flexed to 90°), and the knee of the rear leg bent to a depth when the knee could graze a 5-cm thick cushion placed on the floor. The depth of the squat was visually assessed by researchers and feedback was provided on technique if required. Afterwards, participants recovered the body to the starting position, with a full knee extension of the forward leg and maintaining an erect trunk position. No pause was allowed in the transition phase and the ascent (concentric phase) was encouraged to be performed as explosively as possible. The hip joint of the rear leg was kept at 180° throughout the activity.

### Measurement Equipment and Data Acquisition

All devices used in this study were operated as per the manufacturer’s instructions.

#### Vicon

The Vicon 3D motion system with 12 cameras (sampling rate of 200 Hz) was used as a reference to record the velocity in the sagittal plane during the concentric phase. Prior to each testing session, the capture space was calibrated fully in accordance with a measurement error of < 0.3 mm accepted (Merriaux et al., 2017). Vicon cameras were fixed on tripods and spaced around the periphery of capture space to make sure that each reflective marker placed on the barbell (one placed on the mid and two placed at the end of the barbell) was visible to at least two cameras at all times. The 3D displacements of the markers’ position during each repetition were imported to Nexus 2.5 (Vicon Motion Systems Ltd.) software and filtered using a fourth-order low-pass Butterworth filter with a cut-off frequency at 6 Hz by Matlab-based scripts (Weakley et al., 2023). The cut-off frequency was determined using residual analysis during pilot testing. The start of the concentric phase was defined as the lowest point of vertical displacement, and the end was defined as the highest point occurring after the start. MV was determined from displacement of the concentric phase divided by the time required to complete it (i.e., 200 Hz sampling, each data time was 0.005 s), and the highest value was considered as PV.

#### GymAware

The cable of GymAware was attached to the end of a barbell, aligned with the vertical axis as described by the manufacturer (i.e., perpendicularly to the ground) to determine the displacement of a barbell. The velocity data were obtained instantly in the proprietary app (version 2.8) on an iPad running iOS 15. The system’s built-in algorithms automatically identified the concentric movement phase for each repetition. The GymAware sampled and timestamped the barbell displacement data at 20-ms time points and down-sampled to 50 Hz for analyses.

#### Push

The PUSH Band 2.0 was placed upon the center of the barbell as per manufacturer recommendations. The vertical velocity was computed by integrating the acceleration over time using internal algorithms for start and end calculations in each repetition. MV and PV data were initially recorded at a sampling rate of 1000 Hz. However, for Bluetooth transmission to the proprietary app (version 7.1.0) installed on an iPhone 12 (iPhone, Apple Inc., California, USA), the data were down-sampled to 200 Hz in real-time.

#### My Lift

The My Lift app was installed on an iPhone 12 running iOS 14 which used the smartphone’s camera at a recording frequency of 240 frames per second (fps) at full-HD quality to assess MV. The phone was placed on a tripod at a horizontal distance of 1.5 m, away from the side of the Smith machine, to track the whole movement of each repetition. According to the instructions of the developer, the range of motion (the distance between the height of the barbell at the bottom and the height of the barbell in the final position) of each participant was measured by tape during the first session. Three measurements were taken, and the average value was used in the My Lift app for subsequent analysis. A researcher with experience in slow motion apps followed the developer’s instructions strictly to derive MV from each video. This app allowed a frame-by-frame inspection to select the start and finish of the movement, and thus calculate the duration of activity. The start and finish of every repetition were considered as the first frame in which the barbell started to ascend and the first frame in which the barbell stopped that ascension, respectively. MV was calculated as the distance of the ascent (range of motion by each participant) divided by the duration of the lift.

### Statistical Analysis

All data were presented as mean ± SD in Microsoft Excel and transferred into SPSS (version 26.0; SPSS, Inc., Armonk, NY, USA) and JASP (version 0.8.5.1) for additional analyses. Statistical significance was set a priori at *p* < 0.05.

A linear mixed model was utilized to compare Vicon with other devices. In this analysis, the dependent variables were either MV or PV. The devices were applied as the fixed effect, while participants and loads were included as random intercept effects. The likelihood ratio test was used to verify whether “leg” (i.e., the left leg and the right leg) should be included in the analysis as another fixed effect. In addition, mean differences (MDs), mean absolute error (MAE) and Hedge’s *g* effect sizes were used to compare the differences among devices in this study. Effect size magnitudes of 0.00–0.19, 0.20–0.59, 0.60–1.19, 1.20–1.99, 2.00–3.99, and ≥4.00 were interpreted as trivial, small, moderate, large, very large, and extremely large, respectively (Suchomel et al., 2023).

## Discussion

The primary objective of this study was to assess the validity of three commercial devices: a linear position transducer (GymAware), an accelerometer (PUSH), and a video-based smartphone app (My Lift), in recoding MV and PV during the Bulgarian split squat across a range of intensities from 40% to 90% 1RM. The study findings indicate that GymAware could be used as an alternative to Vicon for assessing valid movement velocity data.

While the validity of GymAware, PUSH, and My Lift have been previously investigated (Balsalobre-Fernández et al., 2018; Banyard et al., 2017; Thompson et al., 2020), to the best of our knowledge, this study is the first one to systematically compare those devices to a 3D motion capture system in recording the movement velocity during unilateral resistance exercises. The results presented herein are consistent with prior studies that have already established the validity of GymAware compared to the 3D motion capture system for bilateral resistance exercises. Banyard and colleagues (2017) compared the GymAware and a laboratory-based testing device during the free-weight back squat exercise. Their research substantiated that GymAware exhibited high levels of reliability (CV < 10%) and validity (*r* > 0.9) in recording both MV and PV across various relative loads (20%, 40%, 60%, 80%, and 100% 1RM). Likewise, Askow et al. (2018) undertook a similar comparison between GymAware and the Qualisys motion-capture system during the back squat at 70% and 90% 1RM. Their findings echoed the aforementioned conclusion, highlighting substantial agreement (ICC > 0.94, *d* < 0.6). The current investigation also shows that GymAware is highly valid, with small MD and trivial to small differences when compared to Vicon.

**Figure 1:**
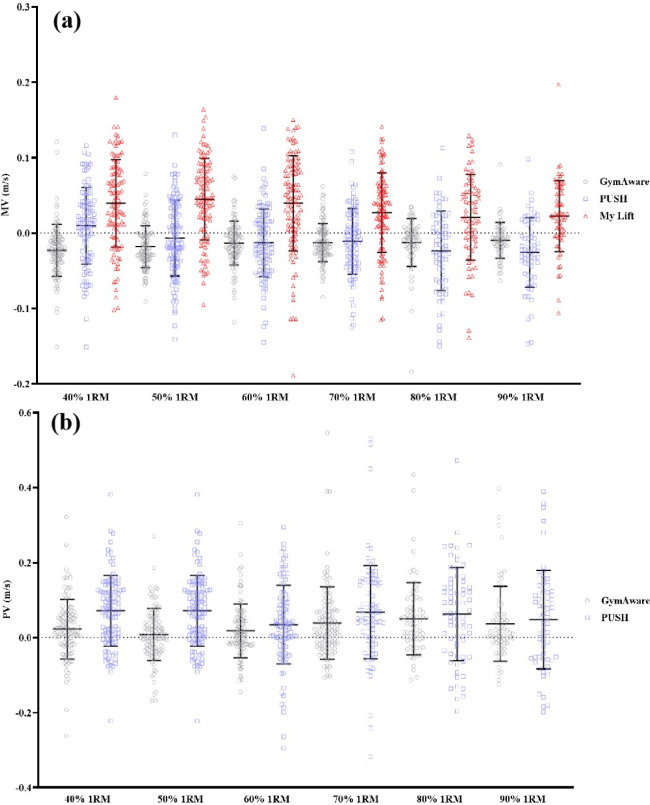
The MD of MV (a) and PV (b) recorded by GymAware, PUSH and My Lift compared to Vicon at specific loads.

With regard to PUSH, while a previous study demonstrated its validity in recording movement velocity on a Smith Machine during the back squat (Balsalobre-Fernández et al., 2016), it is important to note that this study did not use a 3D motion capture system as the “gold standard” and therefore its conclusions may be problematic (Weakley et al., 2021). Recently, a number of studies have further highlighted that the Push Band 2.0, the device used in this study, is not valid for the deadlift (Chéry and Ruf, 2019), the bench press (Orange et al., 2019), and power clean exercises (Thompson et al., 2020), particularly for higher-velocity movements. The findings of the present study further support this conclusion. A comparison between the PUSH and Vicon data revealed significant differences in both MV and PV measurements (MV: *p* < 0.01; PV: *p* < 0.01). As shown in Table 2, the MAE for MV ranged from 0.03 to 0.04 m/s for Push and Vicon, while for PV it ranged from 0.08 to 0.11 m/s. Upon analyzing the data from Figure 1 and taking into account the effect sizes, it is evident that Push tended to overestimate MV as the load increased, with differences ranging from trivial to small. On the other hand, Push appeared to underestimate PV, but only at 50% and 60% of 1RM with a trivial difference. For the other loads, the difference was small, approaching moderate. This noteworthy discrepancy underscores the necessity of cautious consideration regarding the validity of PUSH in PV assessments. This consensus has been reinforced by a recent investigation conducted by Suchomel et al. (2023) who examined the same version of PUSH to record barbell velocity during the jump shrug and hang high pull exercises. They also concluded that the PUSH velocity output was inaccurate due to meaningful differences with GymAware (Suchomel et al., 2023). Thus, based on the evidence provided in both previous research and our findings, we advise coaches against utilizing PUSH for recording athletes’ movement velocity data during unilateral resistance exercises.

**Table 2:** The MAE and Hedge’*g* effect size of MV and PV recorded by GymAware, PUSH and My Lift compared to Vicon at specific loads.

		40% 1RM	50% 1RM	60% 1RM	70% 1RM	80% 1RM	90% 1RM
MV							
GymAware	MAE (m/s)	0.03	0.03	0.02	0.02	0.02	0.02
	*g*	−0.19	−0.19	−0.07	−0.12	−0.11	−0.08
PUSH	MAE (m/s)	0.04	0.04	0.04	0.03	0.04	0.04
	*g*	−0.11	−0.08	−0.06	−0.16	−0.21	−0.22
My Lift	MAE (m/s)	0.06	0.06	0.06	0.05	0.05	0.04
	*g*	0.38	0.59	0.19	0.20	0.18	0.20
PV							
GymAware	MAE (m/s)	0.06	0.05	0.05	0.07	0.07	0.07
	*g*	0.12	0.06	0.06	0.22	0.27	0.20
PUSH	MAE (m/s)	0.09	0.09	0.08	0.10	0.11	0.11
	*g*	0.39	0.18	0.11	0.34	0.33	0.24

Notes: MD = mean difference; MAE = mean absolute error; MV = mean velocity; PV = peak velocity

**Table 3:** Comparisons of MV and PV recordings between Vicon and other devices.

		estimate	SE	t	*p*
MV	GymAware	0.02	0.00	4.79	**< 0.01**
	PUSH	0.01	0.00	3.13	**< 0.01**
	My Lift	−0.01	0.00	−10.21	**< 0.01**
PV	GymAware	−0.03	0.01	−4.79	**< 0.01**
	PUSH	−0.03	0.00	−8.50	**< 0.01**

Notes: MV = mean velocity; PV = peak velocity

In this study, My Lift cannot be recommended due to its significant, trivial to moderate differences (*p* < 0.01, *g* = 0.18 to 0.59) in comparison to Vicon measurements, particularly evident at lower loads (e.g., 40% and 50% 1RM). By contrast, Balsalobre-Fernández et al. (2018) reported excellent validity for My Lift in the bench press (*r* = 0.94, SEE = 0.028 m/s). The inconsistency between their conclusions and ours likely arises from their failure to report the effect size and the 95% CI of the slope and intercept, making it difficult to ascertain the magnitude of the effect size and the potential presence of proportional and fixed biases between the linear transducer and the app. Another possible reason could be the exercise chosen in their study, which focused on the bench press, while the present study focused on the Bulgarian split squat. The variances in range of motion between these exercises could contribute to measurement errors, as noted by Mitter et al. (2021). Notably, while smartphone app developers suggested observer error and misuse may be the main cause, studies such as of Chen et al. (2021) have demonstrated great inter/intra rater reliability (ICC = 0.991 to 0.998) when employing smartphone apps utilizing slow-motion functions. From a practical standpoint, one of the main advantages of smartphone apps should be reduced barriers for operating them in the field. The poor validity of My Lift in this study likely stems from the manual determination of initial and final frames for movement tracking, potentially leading to inaccuracies in identifying the “true” start and end points. Furthermore, it is crucial to acknowledge error sources, including manual range of motion determination and the failure to account for range of motion variability. Even minor discrepancies (such as slight variations in frame selection) can yield substantial outcome disparities. Consequently, this motivates the developers of My Lift to explore methods for accurately determining the precise timing and displacement between the initiation and completion of the movement.

While the findings of this study hold significant implications for coaches and athletes seeking to optimize athletic performance through unilateral VBT, it is imperative to acknowledge the study's inherent limitations. Firstly, only the Bulgarian split squat was investigated, which means the conclusions only pertain to this specific movement. Secondly, this study only explored movement velocity under a fixed trajectory (i.e., Smith Machine). Given the differing movement patterns between free-weight and Smith machine exercises (i.e., free weight movements more closely mimic natural motion by enabling multi-planar motion), the applicability of our findings to free-weight resistance training is somewhat limited. As such, extending the present study’s outcomes to free-weight resistance training should be a priority in future research.

This study is the first to explore the validity among three devices recording MV and PV compared to a 3D motion capture system during a unilateral exercise. We propose GymAware as a valid alternative for coaches seeking to assess MV across full ranges of loads during the Bulgarian split squat. In contrast, Push is a cost-effective product, but cannot be recommended for velocity monitoring due to its poor validity, especially in PV recordings. Similarly, the validity of this device is limited due to manual detection of movement. Therefore, we recommend practitioners to opt for GymAware over Push and My Lift for MV and PV recordings during the Bulgarian split squat.
